# Boosting Performance of Inverted Perovskite Solar Cells by Diluting Hole Transport Layer

**DOI:** 10.3390/nano12223941

**Published:** 2022-11-09

**Authors:** Xiude Yang, Feng Lv, Yanqing Yao, Ping Li, Bo Wu, Cunyun Xu, Guangdong Zhou

**Affiliations:** 1 School of Physics and Electronic Science, Zunyi Normal University, Zunyi 563006, China; 2 Guizhou Key Laboratory for Advanced Materials and Technologies of Clean Energy, Zunyi 563006, China; 3 School of Materials and Energy, Southwest University, Chongqing 400715, China; 4 College of Artificial Intelligence, Southwest University, Chongqing 400715, China

**Keywords:** diluting, PEDOT:PSS, hole transport layer, perovskite solar cells

## Abstract

In our study, by developing the diluted PEDOT:PSS (D-PEDOT:PSS) to replace PEDOT:PSS stock solution as hole transport layer (HTL) materials for fabricating the inverted perovskite solar cells (PSCs), the performance of developed device with ITO/D-PEDOT:PSS/MAPbI_3−x_Cl_x_/C_60_/BCP/Ag structure is enhanced distinctly. Experimental results reveal that when the dilution ratio is 10:1, the optimal power conversion efficiency (PCE) of the D-PEDOT:PSS device can reach up to 17.85% with an increase of 11.28% compared to the undiluted PEDOT:PSS device. A series of investigations have confirmed that the efficiency improvement is mainly attributed to the two aspects: on one hand, the transmittance and conductivity of D-PEDOT:PSS HTL are improved, and the density of defect states at the interface is reduced after dilution, promoting the separation and transmission of charges, thus the short-circuit current (J_SC_) is significantly increased; on the other hand, the work function of D-PEDOT:PSS becomes more consistent with perovskite layer, and the voltage loss is reduced, so that the higher open circuit voltage (V_OC_) is obtained. Our research has indicated that diluting HTL develops a simpler, more efficient and cost-effective method to further improve performance for inverted PSCs.

## 1. Introduction

Recently, organic–inorganic hybrid perovskite solar cells (PSCs) have attracted much attention due to their excellent photoelectric properties and simple preparation methods [1,2,3,4,5]. The efficiency has rapidly soared from initial 3.8% [6] to current 25.7% [7]. Now, PSCs are considered to be the most promising candidate to replace crystalline silicon solar cells for the next generation of commercial solar cells [8]. Generally, efficient PSCs are depended on the complex multilayer structure, in which the perovskite active layer is sandwiched between electron transport layer (ETL) and hole transport layer (HTL). Usually, the perovskite layer is deposited directly above HTL and plays a very important role in determining the device performance.

Poly(3,4-ethylenedioxythiophene):Poly(styrenesulfonate) (PEDOT:PSS) is a conductive polymer with the advantages of high conductivity, high light transmittance, high mobility and easy processing at lower temperatures. Moreover, high-quality PEDOT:PSS films can be obtained by simple coating methods [9,10,11]. In recent years, PEDOT:PSS has most commonly been utilized as an HTL for the inverted PSCs and has achieved favorable device performance [12,13,14,15]. 

However, PEDOT:PSS also has some problems and shortcomings that need to be overcome. For example, it has a low work function and mismatches with perovskite energy level [16,17,18], which is not conducive to the extraction and collection of hole carriers. In addition, the researchers found that the perovskite deposited on PEDOT:PSS were prone to pinholes [19], resulting in poor quality of perovskite films [20,21,22]. Meanwhile, the acidity of PEDOT:PSS will also lead to the instability of device performance. Therefore, many works on the modification of PEDOT:PSS have been reported successively [20,23].

Correspondingly, it is very important and meaningful to further exploit high-performance PSCs via improving the preparation method of PEDOT:PSS HTL. Some researchers have improved the efficiency of PSCs by regulating the work function of PEDOT:PSS. For example, in 2014, Huang et al. [24] improved the device efficiency to 15.4% by regulating the energy level of perovskite and PEDOT:PSS layers. In 2019, Hu et al. [16] modified PEDOT:PSS with sodium citrate. The modified PEDOT:PSS better matched the energy level of perovskite, and the grain became larger, leading to an increase in device efficiency from an initial 15.05% to 18.39%. Some researchers used CsI to modify the interface between PEDOT:PSS and perovskite by reacting with PbI_2_ to form CsPbI_3_, which enhanced the hole transport and extraction in PEDOT:PSS. Therefore, the 20.22% of device efficiency was obtained by this method [18]. 

It has been suggested that the thinner PEDOT:PSS HTL can improve the performance of PEDOT:PSS-based PSCs. In 2018, Sun et al. [14] used H_2_O to wash the prepared PEDOT:PSS film and obtained ultrathin PEDOT:PSS as HTL. The device efficiency could reach up to 18%, while the reference device was only 13.4%. In 2022, Xu et al. prepared an ultrathin PEDOT:PSS HTL on ITO substrate by self-assembly. Compared with the PEDOT:PSS HTL prepared by spinning coating stock solution, the device performance improved from 15.31% to 19.49% [13]. Earlier, in 2016, Snaith et al. [25] added the same methanol solution into PEDOT:PSS stock solution and obtained the highest efficiency Sb-Pb perovskite device at that time. In conclusion, we believe that either rinsing the prepared PEDOT:PSS film with water, or adding the methanol into PEDOT:PSS stock solution or self-assembling the PEDOT:PSS film by immersion, the main purpose is to obtain an ultrathin PEDOT:PSS HTL. However, these processes are complicated and difficult to implement. Is it possible to dilute the concentration of PEDOT:PSS stock solution by adding aqueous solution directly into PEDOT:PSS stock solution, and to prepare ultrathin PEDOT:PSS HTL by simple solution spin coating method to realize high efficiency PSCs?

In our paper, we employed the diluted PEDOT:PSS (D-PEDOT:PSS) realized directly by water to replace the undiluted PEDOT:PSS as HTL materials for fabricating the inverted PSCs, and the device performance with ITO/D-PEDOT:PSS/MAPbI_3−x_Cl_x_/C_60_/BCP/Ag structure is enhanced significantly. It was found that when the dilution ratio is 10:1, the optimal device efficiency can reach to 17.85%, while the undiluted device is only 16.04%. Our research has shown that just after dilution, a thinner PEDOT:PSS layer with a better transmittance, conductivity and energy level can be obtained simply and effectively, and the consumption of HTL material is reduced to one tenth of the original amount, which effectively reduces the preparation cost of PSCs.

## 2. Experiment

### 2.1. Materials

The commercial PEDOT:PSS (4083, 1.3~1.7 wt%), Methylammonium iodide (MAI, 99.9%), lead bromide (PbI_2_, 99.99%), Lead chloride (PbCl_2_, 99.99%), and fullerene (C_60_, >99%) 2,9-dimethyl-4,7-diphenyl-1,10-Phenanthroline (BCP, >99%) were all purchased from Polymer Light Technology Corp (Xi’an, China). N, N-dimethylformamide (DMF, 99.9%) and dimethyl sulfoxide (DMSO, 99.9%) were purchased from J&K Scientific. Metal silver (Ag, 99.99%) was ordered from Tim (Beijing, China) New Material Technology Co., Ltd. and the indium tin oxide (ITO) substrates with square resistance of 14 Ω sq^−1^ used in our experiment were purchased from Advanced Election Technology Co., Ltd. (Yingkou, China).

### 2.2. Preparation of PEDOT:PSS HTLs with Different Dilution Ratios

Commercial PEDOT:PSS (408) is referred to as PEDOT:PSS stock solution in our paper. At the beginning of the experiment, a certain volume of PEDOT:PSS stock solution was diluted with deionized water according to different volume ratios of 1:1, 2:1, 4:1, 6:1, 8:1, 10:1 and 12:1, respectively, to form PEDOT:PSS HTLs, referred to as D-PEDOT:PSS. 

### 2.3. Preparation of Perovskite (MAPbI_3−x_Cl_x_) Precursor Solution

The perovskite precursor preparation has been reported in our previous works [26]. The concentration of 1.4 M was prepared, in which the molar ratio of PbI_2_, PbCl_2_ and MAI is 9:1:10. That is, 580.86 mg PbI_2_, 38.92 mg PbCl_2_ and 222.6 mg MAI are dissolved in 1 mL co-solvent of DMF and DMSO (vol. ratio = 9:1). The precursor solution was placed on a stirring table, and then filtered by PTFE filter head with a pore size of 0.22 μm for later use.

### 2.4. Devices Fabrication

**Figure 1:**
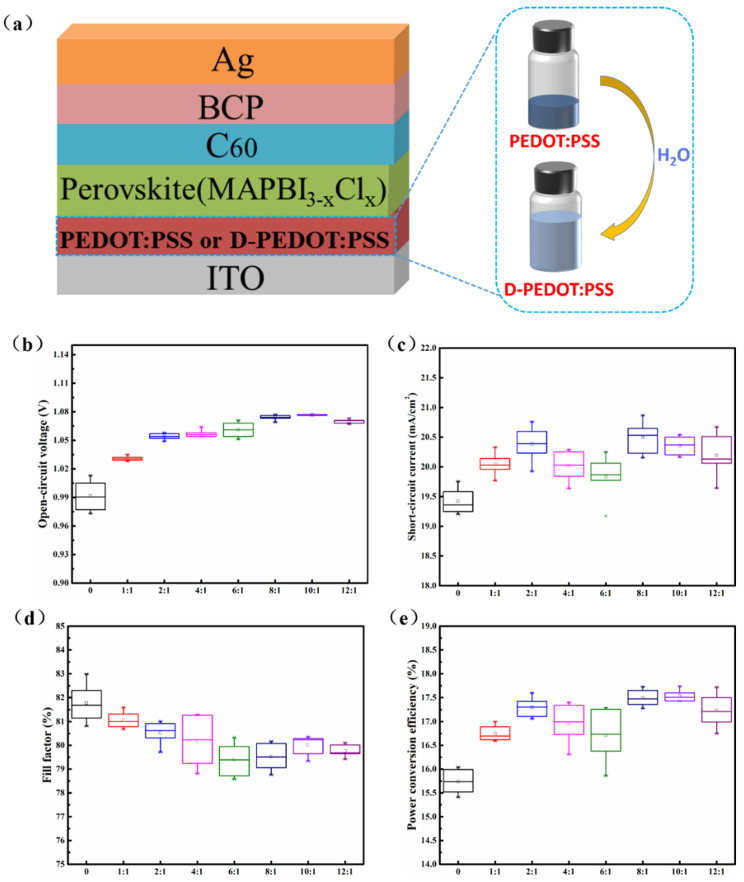
(**a**) Schematic diagram of device structure and preparation of D-PEDOT:PSS hole transport material solution; Photovoltaic parameters of devices with different PEDOT:PSS dilution ratios: (**b**) J_SC_; (**c**) V_OC_; (**d**) FF; (**e**) PCE.

Here, we prepared inverted p-i-n planar PSCs with ITO/PEDOT:PSS (or D-PEDOT:PSS)/MAPbI_3−x_Cl_x_ /C_60_/BCP/Ag structure, which is schematically illustrated in Figure 1a. For comparison, the HTL prepared from PEDOT:PSS stock solution was denoted as the PEDOT:PSS device, and the diluted PEDOT:PSS was denoted as the D-PEDOT:PSS device. 

Firstly, 30 μL PEDOT:PSS or D-PEDOT:PSS solution was spin-coated on the clean ITO substrate at a speed of 6000 rpm for 30 s with an acceleration of 2000 rpm/s^2^, and then placed on a hot plate, at 120 °C, for 20 min. After annealing, it was cooled to room temperature and then transferred into a glove box for depositing perovskite active layer. Next, perovskite precursor was spin-coated on PEDOT:PSS at 4000 rpm for 30 s. During the spin-coating, 200 µL of chlorobenzene (CB) was dropped onto the spinning substrates. Then, the perovskite film was heated, at 50 °C, for 2 min and then 85 °C for 30 min. Finally, 20 nm C_60_, 6 nm BCP and 120 nm Ag electrode were sequentially deposited in high-vacuum using a shadow mask. The cell area defined as the crossing area between ITO and Ag electrode was 0.06 cm^2^.

### 2.5. Characterization

The current density–voltage (J-V) curves were measured by Keithley 2400 source meter in conjunction with 100 mW/cm^2^ (AM 1.5 G) simulated sunlight from a solar simulator (Newport 94043A, Irvine, CA, USA). External quantum efficiency (EQE) was measured in the glove box with a Xenon arc lamp, a monochromatic instrument and a lock-in amplifier. The illumination beam was converged in a small spot using a pre-convex lens, and the size of the spot on the sample is less than the device’s area. The surface morphology, crystal pattern and light transmittance of the as-prepared perovskite films were observed using a field-emission scanning electron microscopy (SEM, JSM-6700F, JEOL, Kyoto, Japan), an X-ray diffraction system (Shimadzu XRD-7000, Kyoto, Japan) and a UV–vis spectrometer (Shimadzu UV-2550, Kyoto, Japan), respectively. 

## 3. Results and Discussion

### 3.1. Effect of Dilution Concentration

To investigate the influence of PEDOT:PSS dilution ratio on the as-prepared devices. The open-circuit voltage (V_OC_), short-circuit current (J_SC_), fill factor (FF) and power conversion efficiency (PCE) were statistically counted, as shown in Figure 1b–e, respectively. It can be seen that V_OC_, J_SC_ and PCE of the device with PEDOT:PSS HTL are lower than the device with the D-PEDOT:PSS HTL. The PCE is the highest when the dilution ratio is 8:1 or 10:1, but device efficiency with the dilution ratio 10:1 is more uniform, and the PEDOT:PSS consumption is less. Accordingly, it can be concluded that diluted PEDOT:PSS HTL plays a positive role in improving device performance. Based on the comparison between the D-PEDOT:PSS device and PEDOT:PSS device, the quality, transmittance, conductivity, photoluminescence, time-resolved photoluminescence, interfacial defect state and work function of perovskite films were investigated comprehensively.

### 3.2. Photovoltaic Performance

**Figure 2:**
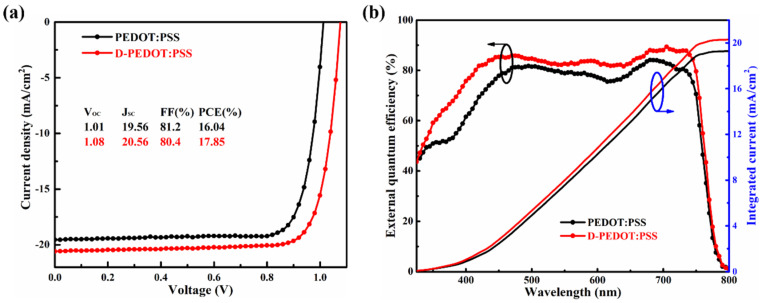
Photovoltaic performance of the optimal devices: (**a**) J−V curves; (**b**) EQE spectrum and integral current.

Figure 2 shows the photovoltaic performance of the optimal devices under 1000 W/m^2^ AM 1.5 G illumination. As seen from Figure 2a, the PEDOT:PSS device exhibits a PCE of 16.04% with a V_OC_ of 1.01 V, a J_SC_ of 19.56 mA/cm^2^, and an FF of 81.2%. The D-PEDOT:PSS device has a PCE of 17.85%, and the V_OC_, J_SC_, FF are 1.08 V, 20.56 mA/cm^2^, 80.4%, respectively. The performance has been significantly improved just use D-PEDOT:PSS replacing PEDOT:PSS as HTL. According to the data of EQE spectrum and integral current exhibited in Figure 2b, the EQE values of D-PEDOT:PSS device is higher than PEDOT:PSS device, and the integral current value is also consistent with the data of the J-V curve. The J-V curves (Figure S1) and EQE spectrum (Figure S2) of D-PEDOT:PSS for each dilution device also confirm the same results.

#### 3.2.1. Characterization of SEM and XRD

**Figure 3:**
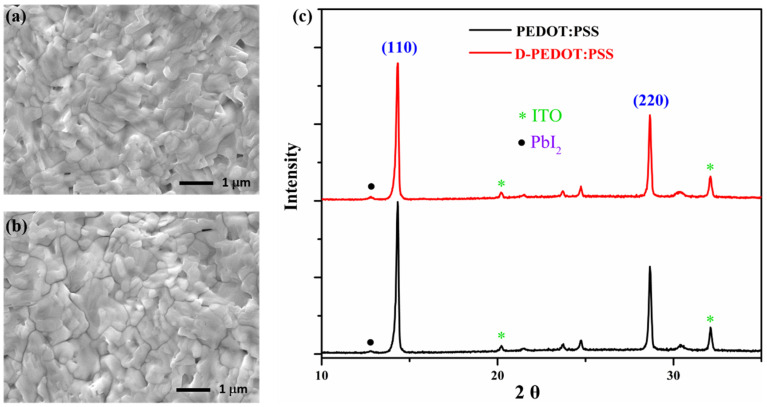
SEM images of perovskite films on (**a**) PEDOT:PSS and (**b**) D-PEDOT:PSS, respectively; (**c**) XRD patterns.

The quality of perovskite films can significantly influence the performance of the device [21,27]. Initially, we thought the morphology and crystallization of perovskite could be improved by spinning perovskite on D-PEDOT:PSS. Accordingly, SEM and XRD were characterized by the morphology and crystallization properties of perovskite films on different HTL substrates. Figure 3a,b are the SEM images of perovskite films on PEDOT:PSS and D-PEDOT:PSS, respectively, showing that there is no significant difference in surface morphology between them, which is further verified by AFM characterization from Figure S3. From the XRD diffraction pattern as shown in Figure 3c, the characteristic peaks are found at 14.14° and 28.48°, corresponding to the crystal plane (110) and (220) of perovskite crystals, respectively. There are no obvious differences in their diffraction intensities and the ratio of two diffraction peaks as well. Similarly, both the absorption of D-PEDOT:PSS (Figure S4) and perovskite films deposited on different D-PEDOT:PSS HTL (Figure S5) have no significant changes. Therefore, we believe that other factors are responsible for the improved device performance. 

#### 3.2.2. Characterization of Optical and Electrical Performance 

**Figure 4:**
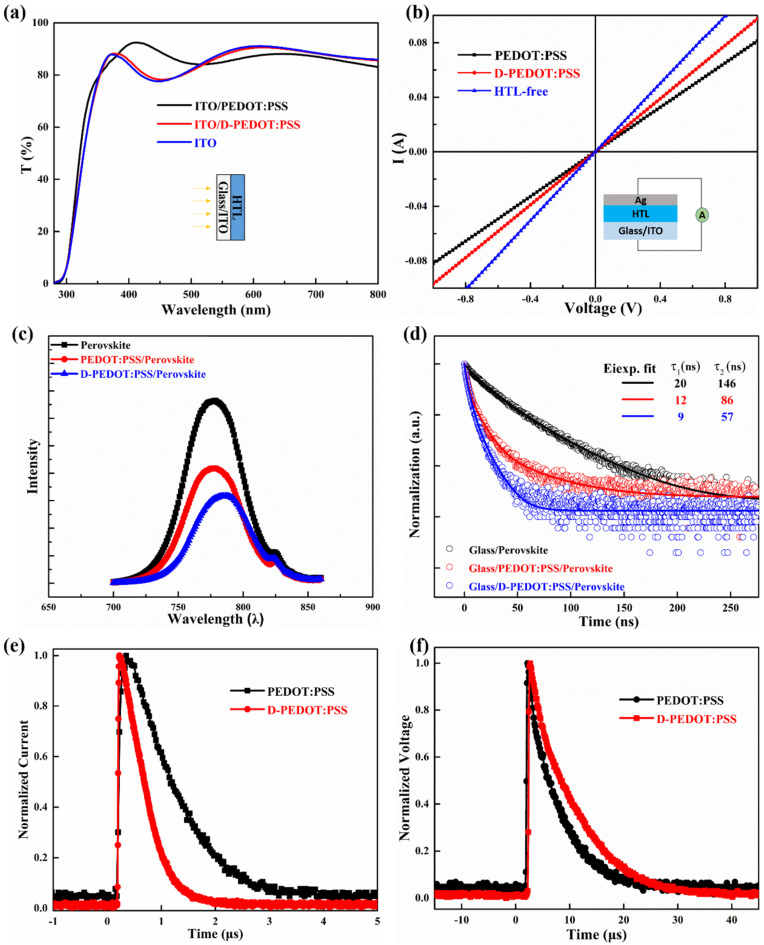
Optical and electrical performance of perovskite films on different HTLs: (**a**) transmittance; (**b**) conductivity; (**c**) PL spectra; (**d**) TRPL spectra; (**e**) TPV; (**f**) TPC.

The use of D-PEDOT:PSS results in a thinner HTL film which will increase the light transmittance. So we guess that the better transmittance of D-PEDOT:PSS will improve the J_SC_ of device. For this, we tested the transmittance of Glass/ITO/PEDOT:PSS, Glass/ITO/D-PEDOT:PSS and Glass/ITO, respectively, as shown in Figure 4a. However, the change in transmittance does not match with EQE variation. Therefore, we consider that the increase in J_SC_ is not only caused by the increase in transmittance. Other reasons for the increase in J_SC_ are the better energy alignment, fewer interface traps, etc. As shown in Figure 4b, we further tested the electrical conductivity of different HTLs. Compared to PEDOT:PSS, the thinner D-PEDOT:PSS indeed shows a higher conductivity, which is conducive to charge extraction and transmission; thus, we believe that this is the main reason for the improvement of J_SC_. 

To further verify above conjecture, the photoluminescence (PL) and time-resolved photoluminescence (TRPL) techniques were employed to study the charge extraction and transmission [28,29]. Figure 4c shows the PL spectra of Glass/Perovskite, Glass/PEDOT:PSS/Perovskite and Glass/D-PEDOT:PSS/Perovskite. Among them, the PL peak intensity of Glass/Perovskite substrate is strongest. With no charge transport layer, the generated electron–hole pairs can only be quenched by competition between radiative recombination and non-radiative recombination. When the perovskite film is deposited on the HTL substrate, the lower peak intensity indicates that the hole is extracted more efficiently at the D-PEDOT:PSS/Perovskite interface, rather than emitting light in the form of radiative recombination in perovskite film with electrons. The PL results mean that PEDOT:PSS is a good hole transport material for hole extraction and the D-PEDOT:PSS is even better. The TRPL experiments were utilized to examine the charge dissociation and recombination process. The bi-exponential fitting I(t) = A_1_exp(−t/τ_1_) + A_2_exp(−t/τ_2_) of TRPL spectra are plotted in solid lines in Figure 4d. The good fitting results mean that there is a fast (τ_1_) and a slow (τ_2_) decay processes in all the three samples. The transmission of photogenerated carriers is believed to be the fast decay process, and the slow decay process is the result of radiative recombination. The TRPL results indicate that the photogenerated holes are extracted and transferred from the perovskite film to HTL more effectively by adding a HTL between perovskite and substrate. The fastest decay of TRPL for the D-PEDOT:PSS sample means the hole extraction and transfer ability can be further improved after diluting with water.

Besides, transient photocurrent (TPC) and transient photovoltage (TPV) were measured to explain the extraction and recombination of charges at the interface. During TPC measurement, the device is in a short circuit state, and the photogenerated charges will be extracted to the external circuit, so TPC can be used to evaluate the transit time of charges across the film after charge generation [30,31]. In Figure 4e, D-PEDOT:PSS device exhibits a shorter TPC lifetime, indicating that the charges can be quickly extracted at Perovskite/D-PEDOT:PSS interface than PEDOT:PSS device. For TPV, it is monitored to reflect the dynamic change in excess carrier recombination process of solar cells [32]. When the pulsed laser is applied on the device, the TPV signal will decay to the original steady-state equilibrium produced by the light bias. The longer TPV lifetime, the longer photogenerated carrier lifetime is. Figure 4f shows that the TPV lifetime of D-PEDOT:PSS HTL is longer, indicating that the photogenerated charge recombination rate in D-PEDOT:PSS device is lower.

#### 3.2.3. Interfacial Defect States

**Figure 5:**
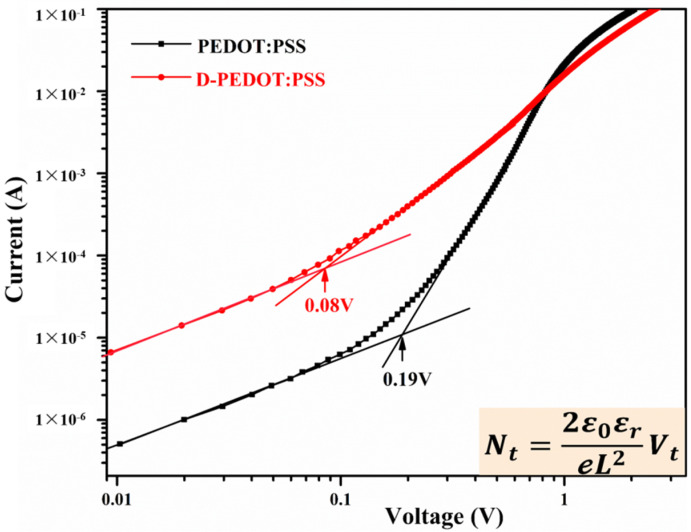
I-V curves of double-HTL devices based on different HTLs.

The longer charge lifetime might be due to the fewer interface states in the device with D-PEDOT:PSS HTL. Here, space charge-limiting current (SCLC) has demonstrated that the low density of defect states at the Perovskite/D-PEDOT:PSS interface is responsible for a longer carrier lifetime. The hole-only devices were fabricated to obtain the density of hole traps using the following architectures: ITO/HTL (PEDOT:PSS or D-PEDOT:PSS)/Perovskite/PTAA/Au for holes. The density of hole trap states (*N_t_*) can be expressed by the following equation [33]:

$$N_{t}=\frac{2\varepsilon_{0}\varepsilon }{eL^{2}}V_{t}$$

where *V_t_* is the filling voltage of the defect states, *L* is the thickness of film, *ε*_0_ is the vacuum dielectric constant, *ε* is the dielectric constant of perovskite films, and *e* is the charge of an electron. Perovskite films are prepared by the same way; we believe that the thickness and dielectric constant in two devices are equal, so the *N_t_* is proportional to the *V_t_*. Figure 5 shows that the *V_t_* of PEDOT:PSS device is 0.19 V, while D-PEDOT:PSS device is 0.08 V. The results indicate that the density of interfacial states becomes lower when perovskite is deposited on D-PEDOT:PSS. The increased J_SC_ is attributed to the higher transmittance of thinner D-PEDOT:PSS HTL and the reduced density of defect states at D-PEDOT:PSS/Perovskite interface, which inhibits the charge recombination and improves the charge extraction efficiency. 

#### 3.2.4. Film Work Function

**Figure 6:**
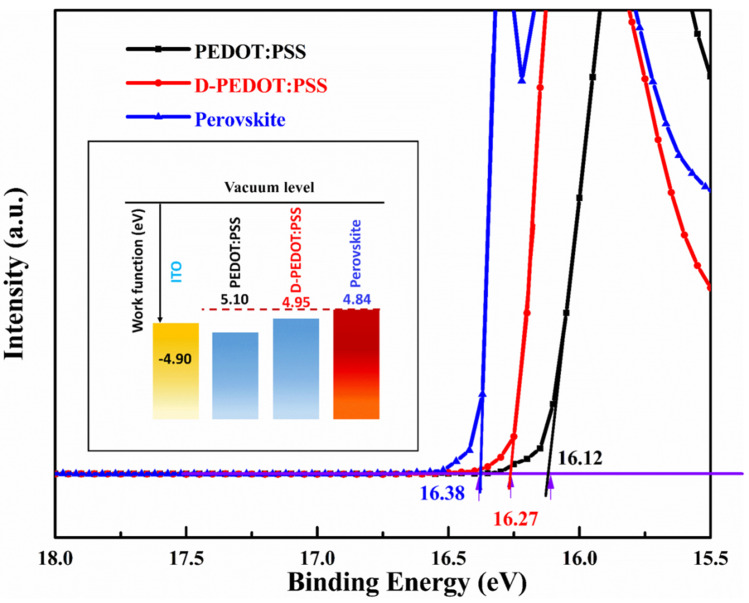
UPS spectra of PEDOT:PSS, D-PEDOT:PSS and perovskite film; the inset is the schematic diagram of energy level structure.

It has been attested that the better energy-level matching between interfaces can effectively reduce the recombination loss [34,35] and obtain the higher V_OC_. As shown in Figure 6, the ultraviolet photoelectron spectra (UPS) of ITO/PEDOT:PSS, ITO/D-PEDOT:PSS and perovskite were tested with the He I (21.22 eV). The secondary electron cutoff edge values of PEDOT:PSS, D-PEDOT:PSS and perovskite are 16.12 eV, 16.27 eV and 16.38 eV, respectively, and the corresponding work functions are 5.10 eV, 4.95 eV and 4.84 eV, respectively. It can be seen from the energy-level structure diagram in the illustration that the work function of perovskite matches D-PEDOT:PSS better than PEDOT:PSS; naturally, the device using D-PEDOT:PSS as HTL can obtain a higher V_OC_.

## 4. Conclusions

In this paper, we developed a series of D-PEDOT:PSS films with different dilution concentrations and PSCs with ITO/D-PEDOT:PSS/MAPbI_3−x_Cl_x_/C60/BCP/Ag structure. The experimental results show that when the volume ratio of water to the PEDOT:PSS stock solution is 10:1, the D-PEDOT:PSS device performance of 17.85% is the best, while the optimal efficiency of the undiluted PEDOT:PSS device is just 16.04%. After dilution, the PCE of developed D-PEDOT:PSS device is enhanced distinctly compared with PEDOT:PSS device. According to a series of characterization, we have concluded that the efficiency increase is mainly attributed to the following two aspects:

(1) Through dilution of PEDOT:PSS, the transmittance and conductivity of D-PEDOT:PSS HTL are improved, and the density of defect states at the D-PEDOT:PSS/Perovskite interface are reduced, which is conducive to the separation and transmission of charges, and thus, J_SC_ is significantly promoted.
(2) According to UPS data, it is found that the work function of D-PEDOT:PSS film is changed, which is more consistent with the perovskite layer, and the voltage loss is reduced, so that the higher V_OC_ is obtained.

In summary, our research provides a simpler and more effective way to further improve the performance of inverted planar PSCs just by diluting HTL, and greatly reduces the consumption of hole transport materials, which effectively saves the cost of device preparation.

## Supplementary Materials

Supporting information can be downloaded at: https://www.mdpi.com/article/10.3390/nano12223941/s1, Figure S1: The J-V curves of D-PEDOT:PSS for each dilution device; Figure S2: The EQE spectrum of D-PEDOT:PSS for each dilution device; Figure S3: AFM image of D-PEDOT:PSS for each dilution; Figure S4: The absorption data of D-PEDOT:PSS for each dilution; Figure S5: The absorption data of perovskite films deposited on different D-PEDOT:PSS HTL.
