# Supplementary material for: Boosting Performance of Inverted Perovskite Solar Cells by Diluting Hole Transport Layer
Source: Nanomaterials (Basel). 2022 Nov 9;12(22):3941. doi: 10.3390/nano12223941 (PMC9699375; doi:10.3390/nano12223941)
Supplement: Supplementary file 1 [file nanomaterials-12-03941-s001.zip › nanomaterials-2008124-supplementary.pdf]

Supplementary materials

Boosting Performance of Inverted Perovskite Solar Cells by Diluting Hole Transport Layer

Xiude Yang [1,2,*,†], Feng Lv [1,2,3,†], Yanqing Yao [1,2,3], Ping Li [1,2], Bo Wu [1,2], Cunyun Xu [3] and Guangdong Zhou [4,*]

[1] School of Physics and Electronic science, Zunyi Normal University, Zunyi 563006, China;
[2] Guizhou Key Laboratory for Advanced Materials and Technologies of Clean Energy, Zunyi 563006, China
[3] School of Materials and Energy, Southwest University, Chongqing 400715, China
[4] College of Artificial Intelligence, Southwest University, Chongqing 400715, China.
* Correspondence: yxiude@163.com (X.Y.); zhougd@swu.edu.cn (G.Z.)
† These authors contributed equally to this work.

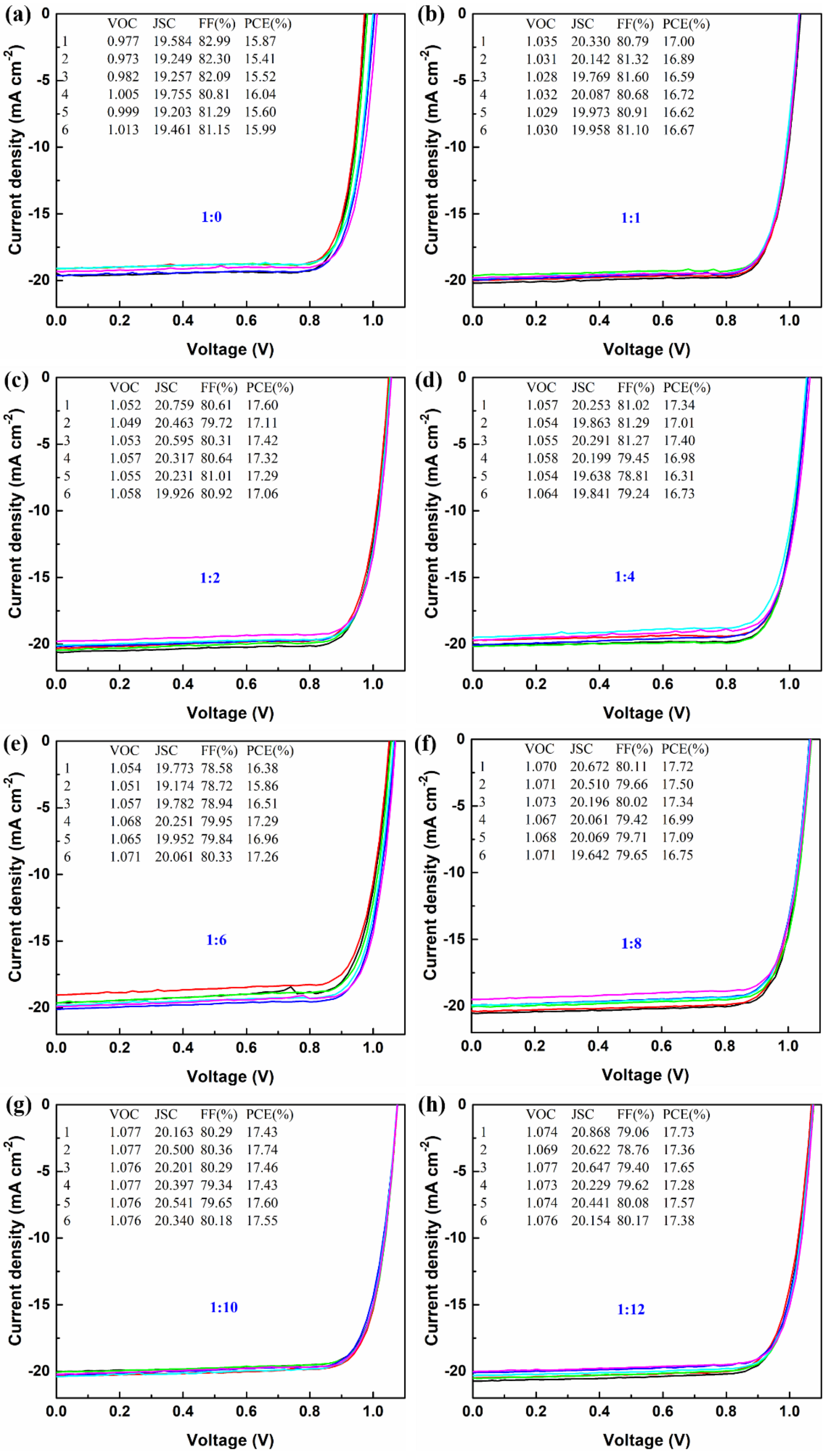

Figure S1 The J-V curves of D-PEDOT:PSS for each dilution device.

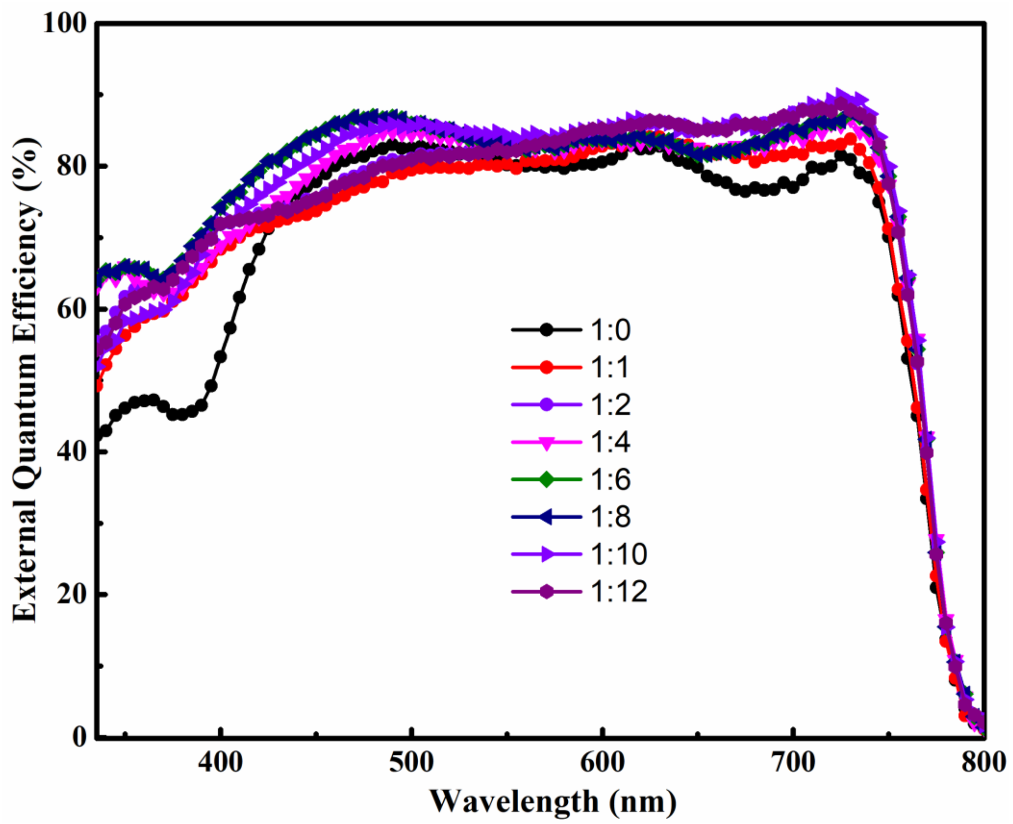

Figure S2 The EQE spectrum of D-PEDOT:PSS for each dilution device.

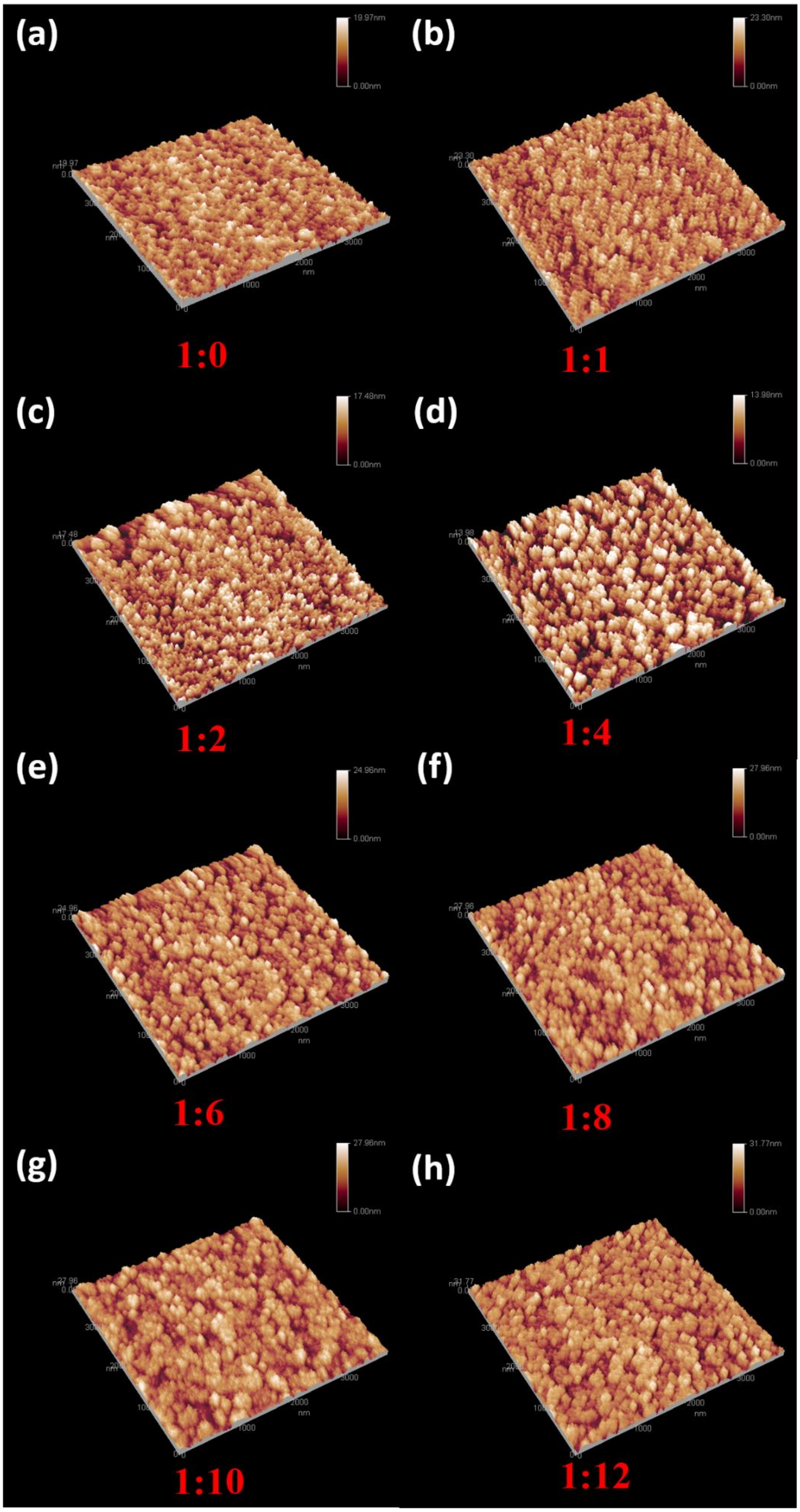

Figure S3 AFM image of D-PEDOT:PSS for each dilution.

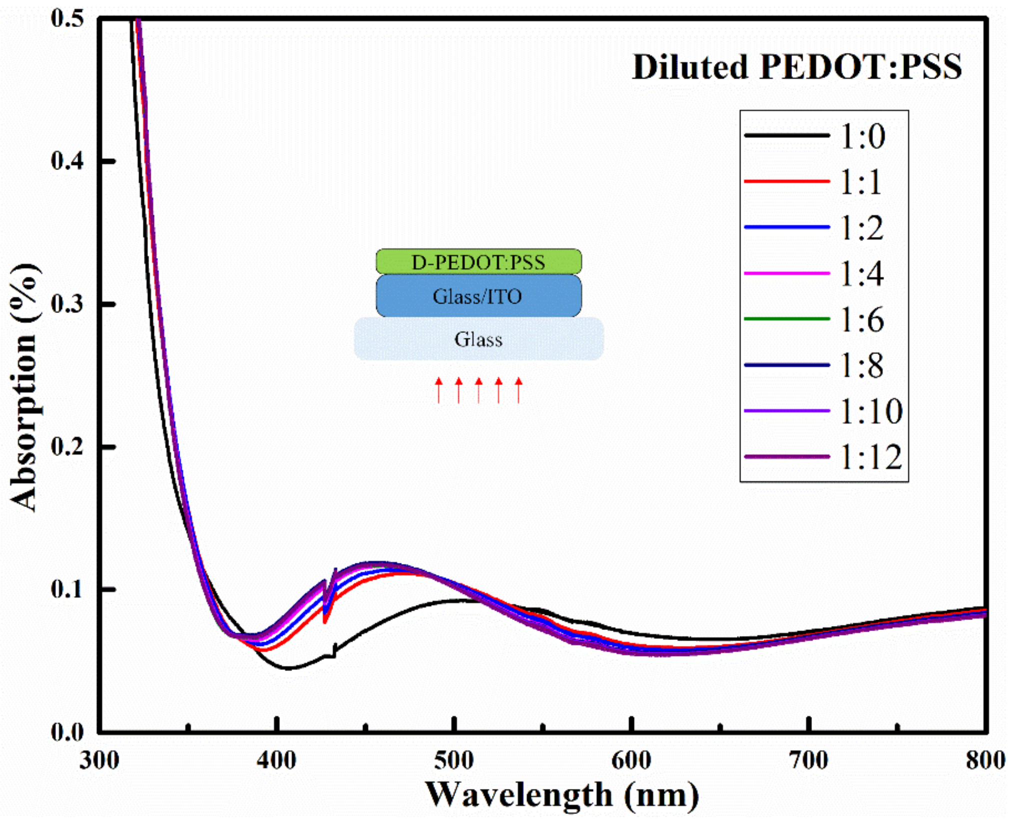

Figure S4 The absorption data of D-PEDOT:PSS for each dilution.

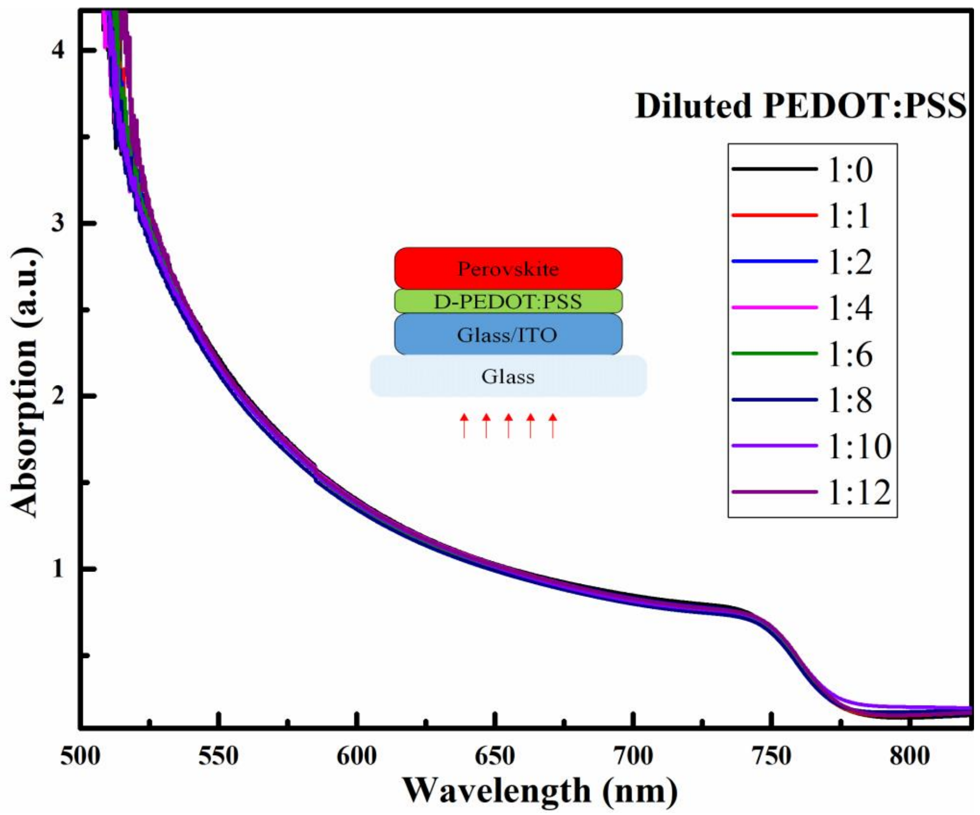

Figure S5 The absorption data of perovskite films deposited on different D-PEDOT:PSS HTL.
